# AUTHOR INDEX

**DOI:** 10.7448/IAS.17.4.19708

**Published:** 2014-11-02

## A

**Table:** 

Abbate, I	P144
Abbrescia, N	P013
Abi-Ghanem, J	P174
Aboulker, J-P	P019
Abouyannis, M	P203
Abrescia, N	P237
Abril, V	P111
Ader, F	P140
Adeyemi, B	P091*
Aebi-Popp, K	P003*
Afonso, C	P294
Ahmad, S	P119
Ahmed, N	P181*
Aiestarán, A	O334
Ajdukiewicz, K	P163
Akalin, H	P063, P146
Akalýn, H	P218
Al Jijakli, A	P117
Alberione, MC	P187
Alberto, T	P136
Albucher, D	P070
Alçada, F	P265
Alcalde Encinas, M	P105
Alcaraz, M	P065
Alcaraz Vidal, B	P105
Aldir, I	P069, P109, P156, P265, P297
Alejos, B	P179*
Alemán, Y	P222
Alexander, H	P199
Alexandra, M	P168
Ali, S	P008
Allan, B	P053
Allavena, C	P044*, P076*, P248*
Almazán, AJ	P105*
Almeida, G	P150
Alongi, I	P079
Altizio, S	P013
Álvarez, D	P222
Alvarez, M	P216
Alves, JP	P109*
Amador, C	P102
Amador, C	P271
Amarsy, R	P100
Amirkhanian, Y	P062*, P086
Ammassari, A	P037, P123, P126, P144, P243, P270, P277, P280
Ananworanich, J	P072
Anderson, J	O133, P001, P098, P162
Andrade Villanueva, J	P022
Andre-Garnier, E	P171
Andrei, C	P159
Andreis, S	P147
Andreoni, M	P147
Andreu-Crespo, A	P077
Angarano, G	P013, P239, P243, P293
Aniekwe, O	P078
Anna, M	P291
Anstett, K	O332
Antela, A	P008, P282, P287
Antequera, P	P065
Antinori, A	O312, O423A*, P037, P039, P041*, P057, P121, P123*, P126, P130, P144, P239, P243, P250, P254*, P261, P267, P270, P274, P276, P277, P280, P293
Antoniadou, A	P210
Antunes, A	P043
Antunes, I	P069, P109, P113, P265
Aragao, F	P247
Aragonés, C	P222
Arama, V	P133
Arasteh, K	P273
Arathoon, E	P251*
Araujo, S	P058
Arazo, P	P104, P271
Arbter, P	P197
Arenas, M	P281
Arenas-Pinto, A	P127
Arkell, P	P203
Arnalich, F	P046
Arribas, J	O423A, P022, P256*, P273*, P275
Arribas, JR	P046
Arribas, JR	P071
Arribas, JR	P132
Arroyo, D	P268
Artero, A	O334
Artioli, S	P180
Asboe, D	P215
Assengone, E	P169
Astuti, N	P227, P244, P266
Atta, J	P116
Atzori, C	P187
Audagnotto, S	P187
Awasom, C	P083
Azevedo, T	P297

## B

**Table:** 

Babiker, A	O112
Back, D	O131, P051, P054
Bader, J	P134
Badura, R	P294
Bagheri, H	P076
Bai, F	P178*, P183, P185
Balestra, P	P123, P126
Ballester Belda, E	P111
Bannert, N	P138
Baño, D	P271
Bañón, S	P042, P058*, P269, P272*
Baptista, T	P069, P109, P113, P156, P265
Baralkiewicz, G	P221
Baran, R	P084*
Barber, T	P030, P162
Barber, TJ	P295
Barceló, Á P065
Barchiesi, F	P120
Barco, A	P185
Barnes, E	P119
Barnes, L	P162
Barralon, M	P219
Barrera, G	P021
Barrufet, MP	P241
Bartmeyer, B	P214
Bartolacci, V	P186
Bartolozzi, D	P250
Basso, M	P147*
Battegay, M	O151*
Baumgarten, A	P106, P112, P209, P217, P224, P238
Bayon, C	P132
Begovac, J	P029
Begovac, J	P016*, P017*
Béguelin, C	P213
Bellacosa, C	P013
Bellagamba, R	P037, P123, P280
Bellazzi, L	P013
Belmonti, S	P259
Beloukas, A	P291
Benatti, S	P244
Benea, O	P133, P159
Benea, S	P133, P159
Benifla, J-L	P100
Beniowski, M	P278
Benirto, C	P111
Benoit, A	P225
Benzie, A	P008
Berenguer, J	P012, P040
Berg, T	P217, P224
Bergmann, J-F	P100
Bernal, E	O334, P191*, P194*, P271
Bernardino, JI	P012, P132, P255
Bernasconi, E	P003, P089
Berno, G	P057
Berthe, A	P100
Bertisch, B	P003
Bertschi, M	P213
Bertz, R	P096
Besutti, G	P128, P184
Bhagani, S	P098
Bhorat, A	P251
Bianco, C	P259, P284, P286
Bicer, C	O423B
Bickel, M	P153
Bickel, M	P045
Bifano, M	P096*
Bigoloni, A	P274, P279
Billaud, E	P044
Bini, T	P041, P178, P183
Biondi, ML	P250
Bisseye, C	P169
Björkman, P	P080
Blanco, F	O334
Blanco, JR	P012, P104
Blasco, AJ	P071
Blasel, S	P045
Blaxhult, A	P029
Blick, G	O222
Bloch, M	P014*
Blonk, M	P257
Bociaga-Jasik, M	P221
Bodasing, N	P160
Boffito, M	P030*, P051, P054, P215, P295*
Bogner, JR	O432B
Boissonnault, M	P233
Boix, V	P111, P271
Bonafont-Pujol, X	P077
Bonczkowski, P	P142, P291
Bonfanti, P	P237
Bonjoch, A	P023, P025
Bonora, S	P039, P187, P239, P267
Boon, G	P231
Booth, C	P220
Borderi, M	P041
Borges, ÁH	O114*
Borges, ÁH	O313
Borges, F	P033, P069, P109, P113, P156, P265
Borghetti, A	P038, P190, P276, P285*
Borghi, L	P178
Borghi, V	P095, P242, P270
Borkar, A	P035
Borland, J	P052
Boschi, A	P242
Bosco, O	P147
Bosse, M	P247
Bossolasco, S	P122, P279
Bottani, GM	P246
Bouee, S	P247
Boulme, R	P219
Boumis, E	P280
Boura, M	P294
Bouzas, MB	P172
Bower, M	O313
Bozzano, F	P186
Bradley, S	P181
Brady, M	P199
Branco, M	P033
Branco, T	P043
Brath, H	P197
Braun, P	P296*
Bravo, I	P018
Bravo, J	P194
Bredeek, F	P261
Brennan, C	O153
Brenner, B	P206
Briggs, A	O211*
Brinkman, K	P004
Brinson, C	O432A, O432B
Brito, J	P202
Brochot, A	P240
Brockmeyer, NH	P010
Brodt, H-R	P045, P188
Brogueira, P	P069*, P156
Brouillette, M	P006
Bruce, D	P096
Brunet, C	P171
Brunetta, J	P225
Bruno, G	P266
Bruyand, M	O235
Bruzzone, B	P186, P250
Bucher, H	P288
Buchholz, F	P174
Buhk, T	P197
Buñuel, F	P065
Burch, L	P001*
Burchard, G-D	P083
Burger, D	P257
Bush Wooley, S	P198
Busi Rizzi, E	P037
Byakika-Kibwika, P	O131
Byrne, L	P161*

## C

**Table:** 

Cabanas, J	P136
Cabié, A	P248
Cagatay, A	P218
Cahn, P	P022*, P262
Caixas, U	P043*, P150*
Calcagno, A	P187*
Caldeira, L	P294
Calistru, P	P093
Callegaro, A	P227*
Callegaro, AP	P244
Callens, S	P291
Calmy, A	P027, P089, P118
Calonge, ME	P139
Calvez, V	P212
Camacho, R	P258
Campbell, NK	P083
Campos, J	P222
Campos, MJ	P109, P202
Cano, A	P074
Cano, A	P191, P194
Capobianchi, MR	P144, P277
Caramello, P	P237
Carbone, A	P243, P279*
Cardona-Peitx, G	P077
Carey, D	O421*
Carganico, A	P106, P217*
Carini, E	P279
Carli, F	P177
Carli, T	P250
Carmena, J	P102
Carosella, S	P246
Carrero, A	P099
Carrero-Gras, A	P287
Carty, C	P231
Carvajal-Molina, F	P132
Casadellà Fontdevila, M	P153*
Casado, A	P282
Casado, JL	P042*, P058, P074, P268, P269*, P272
Cassani, B	P185
Cassetti, I	P015, P084
Cassetti, J	P015
Cassola, G	P049, P186, P239
Castagna, A	P122, P239, P274, P276, P279
Castaño, M	P263, P281
Castelli, F	P237
Castelnuovo, B	P053
Castro, A	P042
Casuccio, A	P079
Cauda, R	P038, P041, P190, P276, P284, P285
Cavassini, M	O153, P003, P089, P090
Caylá, J	O235
Ceausu, E	P093
Ceccherini-Silberstein, F	P267
Cecchini, D	P015*, P172*, P232
Celen, MK	P218
Celesia, M	P013
Celikbas, AK	P218
Cenderello, G	P049, P120, P180*, P186
Ceran, N	P218
Cernuschi, M	P122
Cerutti, B	P020
Ceylan, B	P218
Chaika, N	P062, P086
Chakrabort, D	P174
Chan, D	P015
Chan, K	P131
Chang, B	P225
Chang, L-H	P108, P226
Chang, S-Y	O422, P108, P226
Chaoui, D	P117
Charest, L	P233
Charpentier, C	P212
Charreau, I	P019
Chawki, S	P115*
Cheleboi, M	P020, P134
Chemnitz, J	P174
Cheng, C-Y	P024, P048, P253*
Cheng, S-H	P024*, P048, P253
Cheng, Y-P	P204
Chennebault, JM	P044
Chereau, N	P171
Cheret, A	P248
Cheret, A	P248, P249
Chew, N	P192*
Chidiac, C	P140
Chini, M	P210
Chirianni, A	P013, P041
Choukour, M	P052
Chrysos, G	P210
Chueca, N	P216
Cibea, A	P168
Cicalini, S	P013
Cicalini, S	P037
Ciccarelli, N	P190*, P259, P285
Cicconi, P	P250
Cingolani, A	P121*, P239, P267
Ciraru-Vigneron, N	P100
Clarke, A	O423B*
Cloquell, B	P065
Clotet, B	O153, O423B, P018, P023, P025, P153, P282, P289
Clotet-Sala, B	P077
Clumeck, N	O212*
Co, M	O222
Cogliandro, V	P183
Cohn, S	O131
Colafigli, M	P130, P270, P276, P286
Colby-Germinario, S	O433
Colella, E	P246, P298
Collado, A	P099
Colletti, P	P079
Collins, S	O314, O412*, P098
Colombo, G	P266
Comi, L	P185
Compaore, R	P169
Conesa Zamora, P	P105
Cooper, D	O112
Cooper, DA	O152*
Corbelli, GM	P066
Cordova, E	P232*
Correa, C	P222
Correia AR	P241
Corsi, P	P013
Cortes-Martins, H	P158
Corti, N	P053
Costa, A	P069
Costagliola, D	O113*, P249*
Costantini, A	P293
Costarelli, S	P039*
Cotte, L	P076, P248
Coultas, J	P160
Coutellier, M	P100*
Cozzi Lepri, A	O312, P121
Cozzi-Lepri, A	P039, P153, P205, P239*, P243*, P267, P277, P278, P293
Craig, C	O333
Crauwels, H	P251
Crespo, M	O334, P124
Crespo, M	P282
Cristaudo, A	P130
Crofoot, G	P240
Crouzat, F	P225*
Cruciani, M	P147
Crusells, J	P271
Crusells, MJ	P241
Cuadrado, JM	P065*, P102
Curley, J	P292
Curran, A	P018, P289
Curto, J	P021, P241
Cusini, A	P213
Custodio, JM	P240
Cuzin, L	O113

## D

**Table:** 

Da Silva-Tillmann, B	O222
Dabis, F	O324
Daikos, G	P210
Dalzero, S	P185
D'Amico, R	O222
Dang, N	P008
Darin, K	O131
Darling, K	P090
D'Arminio Monforte, A	O312, P185, P293
D'Arminio-Monforte, A	P039
d'Arminio Monforte, A	O315, O324, P084, P121, P178, P183, P237, P239, P243, P267, P274
D'Arrigo, R	P057*
Daskalopoulou, M	P098*
Dau, L	P275
Davies, O	P199
D'Avino, A	P038*, P190, P285
de Jong, D	P213
de la Tullaye, S	P044
De Leo, P	P186
De Los Santos, I	P101*
De Luca, A	P259*, P267, P276*, P284*, P285, P286, P293
De Maria, A	P186
De Miguel, J	P101
de Oliveira, CF	O153
De Pourcq, JT	P299
De Spiegelaere, W	P142, P291
de Truchis, P	O113
de Wet, G	P170
De Wit, S	O313
De Wit, S	O323, P002
de Wit, S	O324
Deeks, S	O111*
Degen, O	P197*
Degli Esposti, M	P066
Deig, E	P282
del Amo, J	P179
del Arco, A	P263
Del Pin, B	P276
Delaugerre, C	O113
Delforge, M	O323
Delforge, M-L	P002
Delfraissy, J-F	O113
Delgado-Fernández, M	P099
Dellamonica, P	P076
Delobel, P	P019, P248
Demarest, J	O333*
Demaret, J	P140
Demeester, R	P002*, P032*
Demeester, R	P060*
DeMicco, M	P096
Demorin, J	P235
Denneny, E	P193
Dentone, C	P180, P186*
Depatureaux, A	P206*
Descamps, D	P212
Desgrandchamps, F	P115
Dessain, A	P163
Desseaux, K	P264
D'Ettorre, G	P270
Dhande, S	P035
Di Biagio, A	O312, P049, P180, P186, P250, P267, P274, P293*
Di Carlo, P	P079*
Di Cristo, V	P298
Di Filippo, E	P227, P244, P266
Di Giambenedetto, S	P038, P190, P259, P270, P274, P276, P280, P284, P285, P286
Di Matteo, S	P266
Di Nardo Stuppino, S	P246
Di Perri, G	P187, P235, P243
Di Yacovo, S	P289
Diallo, A	P040
Dias, C	P043, P150
Diaz, A	P074, P139, P268
Diaz de Santiago, A	P269, P272
Díaz-Menéndez, M	P099, P287
Díaz-Pollán, B	P189
Dickinson, L	P054*
Dietz, C	P273
Digmann, B	P219
Dilly Penchala, S	O131
Dimi, S	P070
Diniz, A	P158*
Dion, H	P283
Divala, O	P164*
Diz, J	P282
Djigma, F	P169
Doco-Lecompte, T	P118
Dodgen, C	P175
Dokuzoguz, B	P063*
Dolores-Moreno, A-M	P100
Domenico, C	P037
Domingo, P	P241, P282
Domingo-Pedrol, P	P287*
Dona, MG	P130
Dorman, E	P073
Dorner, TE	P067, P068, P197
Dosekun, O	P145
Dragobratovic, A	P016
Dragovic, G	P017, P088*
Dravid, A	P035*
Dretler, R	O434
Drexler, J-F	P083
Dribnohodova, O	P047
Drimis, S	P210
Dronda, F	P074
Dunayeva, E	P047
Dunn, D	O217, P207
Dupke, S	P106
Dupke, S	P217
Duquenne, A	P143
Duro, R	P151, P229
Duvivier, C	P248

## E

**Table:** 

Ecclesia, S	P187
Echevarria, J	O432A, O432B
Echeverría, P	P023*, P025*
Edwards, S	P098, P181
Efremova, O	P047
Efsen, AM	O235*
Ehmer, J	P134
Ehret, R	P112*, P209*, P217, P224
Eisele, L	P010
El Bouzidi, K	P207*
Elbirt, D	P256
Elford, J	O314, P001
Elion, R	O222
El-Sadr, W	O141*, O315, O324
Else, L	O131, O132
Else, LJ	P289
Elzi, L	P089
Encarnación, C	P129
Engelhard, E	P004*
Eraksoy, H	P063, P146
Erdbeer, G	P141, P157
Eron, JJ	O222
Erscoiu, S	P093
Escoriu, S	O434
Escribano Viñas, P	P105
Espinosa, N	P099
Esser, S	P010*
Estany, C	P018
Esteban, H	P124
Esteves, J	P202
Estrada, V	P012*, P189*, P255
Evans, J	P100

## F

**Table:** 

Fabbiani, M	P038, P190, P270, P276, P280, P285
Falasca, K	P120
Fang, A	O333
Fantauzzi, A	P286
Fanti, I	P038, P190
Fanti, L	P259, P284
Farina, C	P227
Farinha, H	P136
Farr, AM	P006*
Fätkenheuer, G	P137
Faturyiele, O	P020, P134
Federico, L	P037
Fehr, J	O423A, P003, P053
Feldt, T	P083
Fenoglio, D	P186
Fenske, S	P141, P157
Fenton, KKL1*
Fernandes, S	P173
Fernandez, P	P282
Fernández-Caballero, J-A	P216
Fernandez Giuliano, S	P172
Ferrea, G	P186
Ferrer, E	P021*, P055, P241, P289
Ferretto, R	P147
Ferry, T	P140
Fialho, R	P097*
Fibriani, A	O154
Fidler, S	O112, P288*
Fiedler, S	P214
Figueiredo, C	P173, P229
Figueroa, MI	P022
Filaci, G	P186
File, A	P097
Fincanci, M	P063, P146
Firlag-Burkacka, E	P201
Fisher, M	O314, P036, P097, P098, P275, P292*
Flamm, J	P261
Flanagan, S	P162*
Flash, C	P198*
Flateau, C	P100
Fletcher, D	P225
Florence, E	P002
Flores, J	O334, P102
Focà, E	P276
Fonsart, J	P019
Fonseca, C	P222
Ford, N	P072
Fordyce, MW	P240
Foster, C	P161
Fowler, N	P203*
Fox, J	O112, P199*, P288
Fraccaro, P	P180, P186
Francisci, D	P120, P276, P286
Fransen, K	P002
Franzetti, M	P298
Frasca, M	P130
Frater, J	P199
Fredrick, L	O222
French, MA	O114
Fuentes-Ferrer, M	P189
Fuertes, R	P202
Fun, A	P223
Furrer, H	O235, P029, P089*
Fuster, MJ	P008
Fux, C	O322
Fýsgýn, NT	P218

## G

**Table:** 

Gabriel, L	P026
Gabrielli, C	P120
Gagliardini, R	P038, P190, P284, P285, P286*
Galanakis, C	P283
Galera, C	P194, P271
Galindo, J	P111
Galindo, MJ	P102
Gallant, J	P235
Galli, L	O312, P122, P274, P279
Galli, M	P237, P246, P254, P298
Gallien, S	P019*
Garaffo, R	P076
Garcia, E	P191, P194
Garcia, F	O334
García, F	P216*
García-Deltoro, M	O334
Garcia-Deltoro, M	P102
Garcia Deltoro, M	P111
Garcia-Diaz, A	P220*
García García, J	P105
Garcia Rodriguez, M	P111
Gargalianos, P	O234, P210
Garimella, T	P096
Garin Escriva, N	P299*
Garlicki, A	P221
Garner, W	P240
Garner, W	P261, P273, P275
Gaseitsiwe, S	P154
Gasiorowski, J	P221
Gaspar, G	P101
Gatell, J	P022, P029
Gatell, JM	O434*
Gatell, JM	P071*
Gatell, JM	P241
Gatey, C	P264
Gathe, J	O222
Gazaignes, S	P264*
Gazzard, BKL3*, P008, P235*
Gazzola, L	P183
Geerlings, S	P004
Geijo, P	P282
Geit, M	P135, P236
Genet, P	P117*
Georgiou, O	P087
Gerasimova, N	P110
Gerbaud, L	P076
Gerbe, J	P117
Gerstoft, J	O423B
Ghafouri, N	P008
Ghisetti, V	P246
Ghosn, J	O113
Giacomelli, A	P298
Giacometti, A	O312
Giacomini, M	P049, P180, P186
Giannetti, A	P037, P126, P270
Giannini, B	P049
Gianotti, N	P122, P254, P267*, P279
Gibbons, K	O222
Giglio, A	P130
Gilleece, Y	P036
Gil-Pérez, D	P104
Gilson, R	P001, P098, P200
Giner, L	P111
Girard, P-M	O423A, P249, P256
Girard, PM	P094, P235
Girardi, E	O235, O312, P121, P144, P237, P277
Gisler, V	P089
Giuliani, M	P130
Givens, N	O153
Goepel, S	P045, P116
Goffard, J-C	P002
Gökengin, D	P063, P146
Goldswain, C	P231
Golics, C	P008
Goliusova, M	P047
Gomes, P	P136
Gomez, C	P021
Gomez, C	P269, P272
Gomez, F	P065
Gomez-Ayerbe, C	P074, P139, P268
Gómez-Berrocal, A	P099
Gomez-Berrocal, A	P228
Gómez-Garre, D	P012
Gómez-Garré, D	P189
Gomez Verdú, JM	P191, P194
Gonzalez, D	P216, P219, P220
González, M	P023, P025
Gonzalez-Baeza, A	P132*
Gonzalez Baeza, A	P046, P127*
González-Doménech, CM	P263, P129
Gonzalez-Garcia, J	P255*
Goodall, L	P160*
Göpel, S	P188
Gordon, M	P252
Gorenec, L	P016
Gorgolas, M	P241
Gori, A	P039, P239, P243, P293
Gori, C	P057
Goubau, P	P002, P143
Goujard, C	P249
Gowers, A	P014
Grabczewska, E	P221
Graham, H	P292
Grarup, J	P040
Gras, L	O154
Grawe, C	P003
Grgic, I	P016
Grilli, E	P276
Grima, P	P190, P276
Grinsztejn, B	P094
Grulich, A	O313
Grzeszczuk, A	P221
Guaraldi, G	P095, P121, P128*, P177, P184, P274
Guardiola, M	P023, P025
Guastavigna, M	P246
Guerra, M	P186
Guffanti, M	P279
Guimard, T	P044
Gulick, R	O431*
Gupta, RS	P020
Gutiérrez, F	O334
Gutierrez, F	P111, P124
Gutierrez-Liarte, A	P228*

## H

**Table:** 

Haas, B	P135, P236
Haberl, A	P045, P116, P188
Habtamu Jemal, Z	P080
Hackmann, K	P174
Hadacek, MB	O423B, P256
Hagins, DP	O434
Hammerl, R	P083
Hamouda, O	P138
Han, Y	O332
Hanna, GJ	O432A, O432B
Haque, T	P220
Harbutly, M	P047
Haro Abad, JM	P299
Harper, K	P231
Harrison, N	P097
Hart, G	O314, P200
Hartmann, P	P197
Harvey, C	O434
Harvey, R	O313
Hasson, H	P122
Hassounah, S	O433
Hatleberg, CI	O324*
Hattingen, E	P045
Hattingh, M	P170
Hatz, C	P020
Hatzakis, A	P210
Hauber, I	P174
Hauber, J	P174
Hauser, A	P138*
He, N	P196
Heera, J	O333
Helleberg, M	P114
Henning, L	P053
Henry, K	O153
Hernandez, J	P124
Hernández Quero, J	O334
Hernández-Quero, J	P268
Hernández-Quero, J	P281, P287
Hernandez-Tamames, JA	P046
Hernandis, M	P065
Hernando, V	P179
Herrero, C	P268
Herrmann, E	P188
Heuhaus, J	O322
Hidalgo-Tenorio, C	P281
Hill, A	O216*, O237, O423A, P030, P051*, P072, P256, P290, P295
Hill, A	O423A
Hill, T	O133, O216
Hillenbrand, H	P238
Hines, P	P028
Hitoto, H	P044
Hlebowicz, M	P221
Hoen, B	P248
Hoesli, I	P003
Hoffmann, C	O434, P141, P157
Hofmann, A	P138
Hofstra, LM	P223*
Holzendorf, V	P010
Horban, A	O423A, P094*, P201
Horst, H-A	P141, P157
Hower, M	P010
Hristea, A	P133, P159
Hu, YB	O222
Huchet, E	P233
Hüe, H	P044
Hung, C-C	O422*, P048, P108*, P226*, P253
Huntington, S	O133*
Hwang, C	P096

## I

**Table:** 

Ianache, I	P093
Iannotti, N	P183*
Iannuzzi, F	P178
Icard, V	P140
Ifekandu, C	P078*
Imaz, A	O334, P055
Immordino, P	P079
Imperiale, D	P187
Inan, D	P063, P146, P152, P218
Inci, A	P218
Ingiliz, P	P106*, P112
Inshaw, J	O112
Ioannidis, P	P087
Iribarren, JA	P124
Irigoyen Olaiz, C	P104

## J

**Table:** 

Jablonowska, E	P221
Jackson, V	O132, P231
Jacomet, C	P076
Jaeger, H	P238
Jagjit Singh, G	P030, P295
James, C	P203
Jankowska, M	P221
Janssen, K	P094
Jansson, PO	P040
Jayewardene, A	P014
Jeanbat, V	P247
Jensen, B	P137
Jensen-Fangel, S	P114
Jerva, F	P052
Jevtovic, D	P017, P088
Jipa, R	P133*
Jipa, RE	P159*
Jochum, J	P083
Johansen, IS	P114
Johanssen, V	O423B
Johnson, M	O314, O322, O424, P001, P098, P220
Johnston, SS	P006
Jones, R	P215
Jose, S	O216, P036*
Joshi, SR	O432A, O432B
Jover, F	P271
Jover Diaz, F	P065
Jover Perez, S	P065
Junge, J	P114

## K

**Table:** 

Kaiser, R	P137, P211, P258*
Kakalou, E	P087*
Kakuda, TN	P240
Kalaria, D	P225
Kaleebu, P	O112
Kalidi, I	P019
Kalli, F	P186
Kambugu, A	P053
Kamele, M	P134
Kamya, MR	P075
Kanatschnig, M	P135, P236
Kandoussi, H	P096
Kanestri, V	P047*
Kann, G	P116
Kaptan, F	P146, P218
Karaoðlan, Ý P218
Karpinski, J	P174*
Karpov, I	O234
Katlama, C	P248
Katzenstein, TL	P114
Kazatchkine, M	O231*
Kegg, S	P245, P275
Keikawus, A	O434
Keller, M	P097
Kelly, D	P061
Kelly, J	P062, P086*
Khatri, A	O222
Khaykin, P	P188
Khokhlova, O	P110*
Khoo, S	O131, O132, P051, P054
Kifaro, E	P154
Kimaro, J	P154*
Kingston, M	P163
Kiragga, A	P260
Kirk, O	O233*, O234, O235, O313, O322, P029, P205
Kiselinova, M	P142, P291
Klauck, I	P028
Klimkait, T	P134
Knechten, H	P296
Knickmann, M	P296
Knobel, H	P282
Knysz, B	O234, P221
Koenig, B	P238
Koeppe, S	P238
Konnov, D	P047
Konnov, V	P047
Koratzanis, G	P210
Kordossis, T	P210
Korten, V	P063, P146*, P218
Kouri, V	P222*
Kourkounti, S	P210
Kouyos, R	P003
Kovacs, C	P225
Kowalska, JD	P040, P201*
Kozirina, N	P047
Kramer, V	O433
Kraus, D	P089
Kravchenko, A	P047, P103*
Kronborg, G	P153
Kroon, F	P004
Krznaric, I	P084, P106, P112
Kücherer, C	P214
Kuecherer, K	P138
Kuimova, U	P047
Kuimova, U	P103
Kulagin, V	O153
Kulkarni, M	P035
Kumar, M	P028
Küpper-Tetzel, CP	P188*
Kusic, J	P017, P088
Kutlu, SS	P218
Kutsyna, G	P029
Kuznetsova, A	P062, P086
Kwirant, F	P010

## L

**Table:** 

Labhardt, ND	P020*, P134*
Lacombe, J-M	O113, P249
Lacombe, K	O221*
Lacor, P	P002
Ladelund, S	P114
Ladisa, N	P013
Ladnaya, N	O236
Lahoulou, R	P248, P249
Lai, C-C	P226
Laker, E	P260*
Lala, MM	O121*
Lalezari, J	O222
Lalezari, J	O432A, O432B
Lama, J	P022
Lamberet, A	P171
Lambert, J	O132*, P231
Lamonica, S	P038, P259, P284, P285
Lamorde, M	O131, P053
Lampe, F	O314, O424, P001, P098
Landovitz, R	P198
Lane, CKL2*
Lanzafame, M	P290*
Lapadula, G	P029, P039
Lapadula, P	P262
Laplante, F	P292
Laquintana, V	P130
Lassandro, A	P038
Lataillade, M	O432A*, O432B*
Lathouwers, E	P094
Latiff, GH	O432A, O432B
Latini, A	P130*, P270, P276, P280
Lattuada, E	P290
Launay, E	P171
Launay, O	O113
Laura, L	P037
Laut, KG	O234*
Lavoie, S	P233, P283
Lavreys, L	P251
Law, M	O315, O322, O324
Lawless, M	O132
Lazanas, M	P210
Lázaro, P	P071
Lazarus, J	O234
Lazzarin, A	O153, P122, P153, P274, P279
Le Blanc, D	O132
Leal, M	P241
Lebech, A-M	P114
Lecompte, MT	P089
Lecompte, T	P003, P027
Ledergerber, B	P135, P236
Lee, K-Y	O422
Lee, V	P059, P107
Lefebvre, E	O433
Legault, D	P233
Legrand, J-C	P032, P060
Leichsenring, B	P067, P068
Leierer, G	P135*, P236
Leipsic, J	P128, P184
Lejone, TI	P020, P134
Lemanska, M	P221
Lengauer, T	P211
Leo, YS	P092, P148
Leone, D	P147
Leszczyszyn-Pynka, M	P155, P221
Letona, S	P104
Li, H-C	P204*
Li, L	P192
Libertone, R	P037, P123, P126*, P144
Ligabue, G	P128, P184
Lim, C	P215*
Lim, R	P192
Limia, C	P222
Limiti, S	P190
Lin, AWC	P131
Lin, S-W	O422
Linder, V	P231*
Linton, N	P014
Liu, H	P235
Liu, W-C	O422, P108, P226, P253
Liu, Y	P292
Llerena, G	P111
Llibre, JM	P241
Llibre, JM	P268
Llibre, JM	P278*
Llibre-Codina, JM	P077*
Lloret, R	P065
Lo Caputo, S	O312, P237, P243
Loiacono, L	P270, P280
Lojewski, W	P221
Lombardi, F	P284
Longpré, D	P233, P283
Looi, E	P163
Lopes, A	P100
Lopez Aldeguer, J	P111
López-Aldeguer, J	P102, P179
López-Cortés, L	P281
Lopez Garcia, B	P299
Lorenzini, P	P037, P123, P126, P237 P270, P277, P280
Losso, M	P278
Losso, MH	O235
Loutfy, M	P225
Lozano, F	P263
Lukas, D	P016, P017
Lundgren, J	O213*, O234, O235, O313, O315, O322, O324, P029
Lundgren, JD	O114, P153, P205
Luo, W-L	P096
Luo, Y-Z	O422, P048, P108, P226, P253
Lutz, T	P238
Lye, D	P148
Lynen, L	P020

## M

**Table:** 

Machouf, N	P233, P283
Machuca, I	P058
Maciejewska, K	P155, P221
Mackie, N	P145, P207
Madeddu, G	O312*, P039, P267
Madeddu, G	P276
Madruga, JV	P084
Maffongelli, G	P147
Magenta, A	P183
Maggi, P	P013*
Maggiolo, F	P008, P039, P227, P239, P243, P244*, P266*, P280
Magiorkinis, E	P210
Magnuson, D	P198
Maillard, M	P122
Maillard, M	P279
Malatinkova, E	P142*, P291
Malheiro, L	P151
Maltez, F	P278
Maltezos, E	P210
Mambule, I	P260
Man, P	P128
Mancusi, D	P041, P254
Manea, E	P133, P159
Mangues Bafalluy, MA	P299
Mansinho, K	P033, P069, P109, P113, P136, P156, P265, P297
Mantia, E	P186
Manuel, M	P129
Marcelin, A-G	P212*, P256
Marchetti, G	O312, P183, P185, P243
Marchetti, GC	P178
Marchou, B	P019
Marcotullio, S	P121
Marcus, R	P193*
Mardarescu, M	P159
Märdärescu, M	P168*
Margot, N	P240
Maria Miro, J	O112
Marimuthu, K	P092
Marín, I	P191, P194
Marinho, A	P043, P150
Mariño, A	P124
Markowicz, S	O323*
Marques, M	P043
Márquez, M	P281
Marras, F	P186
Martinelli, C	P013
Martínez, E	P012
Martinez, M	P084
Martinez, M	P172
Martinez, O	P194
Martinez-Colubi, M	P074
Martinez-Colubi, M	P139
Martinez de Tejada, B	P003
Martinez-Dueñas, L	P268
Martínez Fernández, L	P105
Martínez Madrid, O	P105
Martínez-Madrid, O	P271
Martínez-Madrid, OJ	O334
Martinez-Tristani, M	P081
Martini, F	P144
Martorell, C	P292
Martynenko, O	P061*
Martynenko, O	P085
Maruapula, D	P154
Mary-Krause, M	O113
Masegosa, C	P065
Maselle, E	P075*
Maserati, R	P013
Masiá, M	P102
Masini, G	P244
Maso, M	P055
Masse, V	P117
Mastroianni, C	P243
Matei, C	P168
Matheron, S	P100
Matthes, N	P027
Mattioli, MI	P015
Mattioli, S	P066*
Maucort-Boulch, D	P140
Maylin, S	P100
Mayorga, M	P263
Mayorga, MI	P281
Mayr, C	P197
Mazeron, M-C	P100
Mazurek, R	P221
Mazzarello, G	P049
Mazzetti, M	P120
Mazzocato, S	P120
Mazzola, G	P079
Mazzotta, E	P120
Mbisa, JL	P207
McColl, D	P235
McCormick, A	P220
McDonald, C	P292
McDonald, G	O132
McDonnell, J	P098
McFaul, K	P215
McGowan, J	O314*
McQuillan, O	P163
Medina, P	P021
Medstrand, P	P080
Meini, G	P259
Meintjes, G	O311*
Meixenberger, K	P214*
Mekprasan, S	P208
Mena, A	P042
Ménard, A	O113
Mendão, L	P202
Mengoli, C	P147
Menichetti, S	P123, P126
Menozzi, M	P177*
Mensforth, S	P160
Mera Giler, R	P198
Merino, D	P099
Merino, E	P271
Merino, MD	P281
Merkley, B	P225
Merlano, C	P049
Merlini, E	P178
Merry, C	O131
Merz, L	P089
Merz, L	P090*
Mesplede, T	O332
Mesplède, T	O433, P206
Messiaen, P	P142, P291
Metzner, K	P083
Meyer, M	P175
Meyer, RD	O333
Meylakhs, A	P062, P086
Michau, C	P044
Micheli, V	P246
Michelo, C	P164
Mikkola, V	P137
Milinkovic, A	P127
Miller, R	O235
Mineo, M	P079
Miners, A	O314
Minguez, C	P102*, P111
Mínguez-Gallego, C	O334
Miranda, A	P069, P297*
Miranda, AC	P109, P113
Miranda, AC	P156*, P265
Miro, J	O235
Mironov, K	P047
Mitsura, VM	P278
Mocroft, A	O234, O235, O313, O315, O322*, O324, P029*, P278
Moecklinghoff, C	O423A, O423B
Mohrmann, G	P125*
Moioli, MC	P267
Moisi, D	P206
Molina, J-M	O153*, P019, P115, P264
Molto, J	P289
Mondi, A	P038, P190, P259, P276, P285
Moneti, V	P156, P297
Monge, S	P012, P046
Mongiat-Artus, P	P115
Moniz, P	P265*
Monneret, G	P140
Monno, L	P121, P237
Montefiori, M	P049
Monteiro, E	P043
Monteiro, E	P150
Monteiro, N	P033*
Montenegro, N	P173
Montero, M	P102, P111
Montes, M	P094, P101
Montes-Ramirez, M	P132
Montlahuc, C	P115
Moorthy, V	P028
Morales-Ramirez, JO	O434
Morand-Joubert, L	P076
Moranne, O	O322
Moreira, AC	P195
Morello, J	P150
Moreno, A	P042, P058, P074, P139, P268, P269, P272
Moreno, J	P271, P287
Moreno, S	O234, P042, P058, P074, P139, P268, P269, P272
Moreno, V	P255
Moreno Zamora, A	P012
Moretto, D	P130
Morgand, M	P100
Morlat, P	O322
Morosi, M	P298
Moroti, R	P159
Morrison, C	P288
Mosti, S	P037
Motlatsi, MM	P020, P134
Moucho, M	P173
Mouly, S	P100
Mourah, S	P019
Moutschen, M	P002
Moyle, G	P030, P292, P295
Mozer-Lisewska, I	P221
Mudrikova, T	P223
Muga, R	P288
Mugomeri, E	P149
Muhanguzi, A	P075
Muhlbacher, A	P008
Muhumuza, S	P075
Mulcahy, F	P084
Munier, A-L	P264
Muñoz, A	P191, P194
Muñoz, J	P282
Munoz, M	P233
Muñoz-Rodríguez, J	P287
Muriel, A	P074, P139
Murray, M	P008*
Murungi, A	O423B
Musaazi, J	P053, P260
Musatov, V	P062, P086
Muser, J	P020
Musonda, R	P154
Mussini, C	P095, P128, P177, P184, P237, P239, P254, P293

## N

**Table:** 

Nakalema, S	O131
Nakijoba, R	P053
Nalwanga, D	P053, P260
Namusobya, J	P075
Nansubuga, J	P075
Nassimi, N	P083
Navarro, A	P021
Navarro, J	P018
Navarro, V	P271
Navas, E	P139
Nawavvu, C	P075
Ndungu, T	P252
Neagu-Drãghicenoiu, R	P168
Necsoi, C	O323
Negredo, E	P018, P023, P025
Neifer, S	P209
Nelson, M	P030, P094, P180, P205, P295
Nemeth, J	P089
Neukam, K	P099*
Neumann, T	P010
Neves, I	P241
Newell, M-L	O133
Ng, L	P198
Ng, OT	P148
Nguyen, T	P273, P275
Ngwira, B	P164
Nica, M	P093
Nicastri, E	P123
Nicolaescu, O	P093
Niculescu, I	P133, P159
Nieuwkerk, P	P004
Nijhuis, M	P223
Nina, J	P109, P265
Niubo, J	P055, P289
Nkhoma, E	P028
Noah, C	P125
Noguera Julian, M	P153
Norton, M	P022
Norton, M	P081
Nozza, S	P008, P122, P267, P274
Nuñez, C	P065
Nwokolo, N	P215
Nyombi, B	P154

## O

**Table:** 

Obel, N	O235, P114
Obermeier, M	P112, P209, P217, P224*
Ocampo, A	O334, P282
O'Connell, R	P001, P193
O'Connor, J	O424*
O'Connor, JL	O114
Oddershede, L	O217
Okware, S	P053
Olczak, A	P221
Oliveira, M	O332, P206
Olivier, D	P149*
Olson, A	P288
Oomeer, S	O112, P145
Oprea, C	P093*
Opsomer, M	P240, P251
Orcamo, P	P049
Orchi, N	P144
Ordovas, R	P065
Oreni, L	P298
Oreni, ML	P246
Ormaasen, V	P278
Orofino, G	P186, P246, P276, P286
Ortega, E	P102
Ortega, P	P065
Ortega Gonzalez, E	P111*
Ortiz de la Tabla, V	P065
Osiyemi, O	O434
Oteo, JA	P179
Ouermi, D	P169
Ouwerkerk-Mahadevan, S	P094
Owasil, J	P116

## P

**Table:** 

Pace, M	P199
Palacios, R	P263
Pallavicini, F	P038, P285
Palù, G	P147
Panagopoulos, P	P210
Pandolfo, A	P185
Panteleev, A	O235
Panton, L	O214*
Papadopoulos, A	P210
Papanikolaou, K	P087
Paparizos, V	P210
Papastamopoulos, V	P087, P210
Paraskevis, D	P210*
Parczewski, M	P155*, P221*
Paredes, R	O334*, P018*, P153, P205, P278
Parienti, JJ	P008
Parisi, SG	P147
Parkes-Ratanshi, R	P260
Parodi, A	P186
Parruti, G	P120*
Parsons, V	P200*
Pasa, A	P180
Pasquau, J	O334, P124, P281*, P282
Paszkowski-Rogacz, M	P174
Patel, A	O132
Patel, K	P261
Patel, P	P180
Paton, N	O217, P256
Patterson, P	P022, P262*
Pearson, P	P181
Pedersen, C	P278
Pedersen, G	P114
Pedreira, JD	P042
Pellizzer, G	O312
Penco, G	P250
Peng, S	P073
Pereira, B	P076
Pereira, K	P113*, P136
Pereira, M	P097
Pereira, S	P043, P150
Peres, S	P033, P069, P109 P113, P156, P265, P297
Perez, A	P191, P194
Perez, F	P065
Pérez, H	P262
Pérez, J	P222
Pérez, L	P222
Pérez, N	O334
Perez-Caballero, L	P101
Pérez-Camacho, I	P099
Pérez-Elías, MJ	O334
Perez-Elías, MJ	P042, P058, P269, P272
Pérez Elías, MJ	P074*, P139*, P268*
Pérez Elías, P	P074, P139
Pérez-Hernández, IA	P129*, P263*, P287
Perez-Martinez, L	P104*
Pérez-Martínez, L	P271
Pérez-Molina, JA	P124
Perez-Valero, I	P132
Pérez Valero, I	P046*
Pérez Valero, I	P124*
Peri, M	P183
Perno, CF	P057, P277
Perovic Mihanovic, M	P017
Perpoint, T	P140
Perry, N	P098
Peters, S	P090
Petre, C	P168
Petrea, S	P168
Pett, S	O114
Pfeifer, N	P211
Pfeiffer, K	P020
Phillips, A	O314, O424, P001, P098, P153, P207, P288
Phillips, AN	O114
Phillips, AN	P205
Phiri, S	P026
Pialoux, G	O113
Piccoli, B	P038, P285
Pieralli, S	P066
Piérard, D	P002
Pierro, P	P144
Pietra, V	P169
Pietraszkiewicz, E	P201
Pilatus, U	P045
Pillay, D	O133, P205
Pillay, SK	P252*
Pineau, S	P044
Pineda, JA	P099
Piñeiro, C	P173, P151, P229
Pinnetti, C	P037*, P123, P126, P144*, P270, P277*, P280*
Pinto, N	P202
Piolini, R	O312
Piontkowsky, D	P235, P247, P261
Pironti, A	P211*
Pisabarro, MT	P174
Piso, RJ	P234*
Pitisci, L	P032
Plazzi, M	P270
Plazzi, MM	P123, P126
Plettenberg, A	P141, P157
Ploussard, G	P115
Podlekareva, D	O235
Podzamcer, D	P268
Podzamczer, D	P018, P021, P055, P241*, P282*, P289*
Poizot-Martin, I	P248
Pokrovskaya, A	O236*, P047
Pokrovskiy, V	O232*, P110
Poli, A	P122
Policarpo, S	P195
Pontani, D	P273
Popova, A	O236, P047
Porter, K	O112, P200, P288
Portilla, J	P111
Portilla, J	P271*
Post, F	O235, O321*
Poveda, E	O334
Pozniak, A	O216, O237, P051, P205, P207, P215, P261
Pozniak, AL	P040
Pradier, C	O324
Prati, F	P242
Prinapori, R	P286, P293
Pryluka, D	P262
Psichogiou, M	P210
Pucci, L	P057
Pucillo, LP	P057
Puga, D	P134
Pugliese, P	P248
Puig, J	P023, P025
Puls, R	P050
Puro, V	P144
Putcharoen, O	P208
Puzzolante, C	P095*

## Q

**Table:** 

Quan, D	P014
Quashie, P	O332, P206
Quereda, C	P139, P272
Quirino, T	P254, P293
Quiros Roldan, E	P121
Quiros-Roldan, E	P276

## R

**Table:** 

Radoi, R	P093
Raffi, F	P040, P044
Raffi, F	P171, P248
Raggi, P	P184
Rakhmanova, A	O234
Ramgopal, M	P292
Ramjee, G	O112
Ramoeletsi, M	P134
Ranin, J	P088
Rappold, M	P135, P236*
Rauch, A	P213
Ravn, L	O313
Rawlings, K	P198
Raymond, A	O237*
Raymond, Y	P083
Reepalu, A	P080*
Reeves, I	P275*
Reinsch, N	P010
Reiss, P	O234, O315, O322, O324, P004, P029
Reizis, A	P110
Reliquet, V	P044
Reliquet, V	P171*
Resche-Rigon, M	P264
Reus, S	P102, P271
Rhee, M	P235, P292
Ribalta, J	P023, P025
Ribas, Mà, O334
Ribera, E	P018
Ribero, A	P124
Ricbourg, A	P100
Rickenbach, M	O324, P003
Ricottini, M	P123, P126
Rieger, A	P135, P236
Rigo, F	P290
Rijnders, BJA	O154, P257
Rijo, J	P136
Ríos, MJ	P099
Ríos, MJ	P281
Ripamonti, D	O423B
Ristola, M	P278
Ritchings, C	P006
Rivero, A	P094, P263, P268
Rivero, M	P104
Rivero-Juárez, A	P099
Rizzardini, G	P273, P293
Rizzi, M	P227, P244
Roberts, L	P203
Robinson, N	P199
Rocha, LM	P202*
Rocha Pereira, N	P151, P229
Rockstroh, J	O234
Rodger, A	O314, P001, P098
Rodrigues, T	P195
Rodriguez, C	P172, P232
Rodríguez de Castro, E	P282
Rogalska, M	P221
Rogatto, F	P235, P247*
Roger, P-M	P248
Rojas, J	P202
Rojas, JF	P241
Rokx, C	O154*, P257*
Rolls, S	P193
Rolón, MJ	P022
Romito, A	P187
Ronchetti, L	P130
Ronot Bregigeon, S	P008
Rönsholt, FF	O114
Rosario, P	P129
Rosenblatt, L	P006, P073*
Rosi, A	P250
Ross, A	P091
Ross, M	O322
Rossetti, B	P259, P284, P286
Rougemont, M	P118
Rovati, M	P185
Rozas, N	P021, P055, P241, P282
Rozenbaum, W	P264
Rozera, G	P144
Ruane, PJ	O222
Rubio, P	P111
Rubio, R	P124, P179
Rubio, T	P271
Rudin, C	P003
Rudolph, R	P010
Ruelle, J	P143*
Rugina, S	O434
Ruiz Belmonte, E	P105
Ruiz-Morales, J	P099
Rusconi, S	P246*, P274, P276, P286, P298*
Rusted, J	P097
Ruta, S	P093
Ruxrungtham, K	P072, P208
Rymer, W	P221
Ryom, L	O315, O322, O324, P029

## S

**Table:** 

Sabin, C	O133, O315, O324, O424, P036, P205
Sabranski, M	P125, P141*, P157*
Saez, C	P228
Sagna, T	P169
Sahly, H	P125
Saillard, J	P040
Saison, J	P140*
Saiz-de-la-Hoya, P	P271
Sala-Piñol, F	P077
Salemovic, D	P088
Salpietro, S	P279
Samarawickrama, A	P036
Samarina, A	P084
Sambatakou, H	P210
San Román-Montero, J	P189
Sandler, I	P225
Sandrine, O	P277
Sanjoaquin, I	P104
Santoro, A	P128, P177, P184
Santos, AS	P173, P229
Santos, C	P074, P139
Santos, I	P228
Santos, J	P012, P129, P263
Santos, J	P124
Santos, JR	P018
Santos-Hoevener, C	P138
Sanvisens, A	P179
Sanz, J	P124, P228
Sanz, M	P065
Sanz-Moreno, J	P101
Sanz-Moreno, J	P287
Sanz-Sanz, J	P101
Saracino, A	P237*
Sarcletti, M	P135, P236
Sargýn, F	P218
Sarmati, L	P147
Sarmento, A	P151, P173, P229
Sasadeusz, J	P094
Sasse, A	P002
Saumoy, M	P018
Saumoy, M	P021, P055
Savoldi, A	P183
Saxon, C	P119*
Say, V	P061
Sayada, C	P219*, P220
Sayan, M	P152*, P218*
Scaggiante, R	P147
Scaglioni, R	P128
Scarsi, K	O131*
Schalk, H	P197
Schapiro, J	O331*
Schechter, M	O112
Scherrer, A	P053
Schewe, K	P141, P157
Schipper, P	P223
Schmid, P	P089
Schmiedel, S	P083
Schneider, M	P223
Schnepf, N	P100
Schröck, E	P174
Schrooten, Y	P222
Schuettfort, G	P045*
Schuetze, M	P106
Schulte-Hermann, K	P067*, P068*
Schülter, E	P258
Schultze, A	O235, P205*
Schulze, C	P010
Schutten, M	O154
Schwarz, B	P010
Schweitzer, F	P137*
Schwimmer, C	P040
Scognamillo, M	P079
Scott, G	P082*
Scott, J	P034
Sculier, D	P027*, P118*
Sculpher, M	O217
Sedlacek, D	P153
Segura, F	P179
Sekaggya Wiltshire, C	P053*
Sellier, P	P100
Semitala, FC	P075
Serebrovskaya, L	P110
Serna Ortega, PA	P227
Serrano, J	P191, P194
Serrão, R	P151*, P173*, P229*
Setti, M	P049, P180
Sevgi, DY	P218
Shahgildyan, V	P047
Shao, E	P154
Sharma, JR	P175*
Shepherd, L	O313*
Sherr, L	O314, P001, P098
Shivaji, T	P158
Siccardi, M	P054
Sierra Madero, J	P022
Sighinolfi, L	P242
Signori, A	P186
Signorovitch, J	P005
Silaghi, R	P251
Silva, J	P136*
Silverberg, M	O313
Simmons, B	P072*
Simon, F	P100
Simoneau, G	P100
Simpore, J	P169
Sin, D	P128
Sin, DD	P184
Singh, A	P252
Singh, U	P252
Sirivichayakul, S	P208
Skepu, A	P175
Skogmar, S	P080
Skoutelis, A	P087, P210
Skrahin, A	O235
Skwara, P	P221
Slim, J	O222
Smilg Nicolás, C	P105
Smith, C	O315*, O322, O424, P001, P004, P088, P098
Smith, G	P225
Soares, J	P151, P229
Sobrino, P	P012, P179
Socías, E	P262
Sofia, S	P013
Somogyi, S	P214
Sonecha, S	P215
Song, I	P052*
Song, J	P005
Sönnerborg, A	P258
Sonntag, I	P141, P157
Sorensen, S	P073
Soto, K	P150
Soto, Y	P222
Souak, S	P100
Soubeiga, ST	P169*
Sozio, F	P013
Spagnuolo, V	P122*, P274*, P279
Speakman, A	O314, P001, P098
Speight, C	P026
Squillace, N	P041
Staehelin, C	P003
Stefanek, W	P067, P068
Steininger, K	P106
Stellbrink, H-J	P125
Stellbrink, H-J	P141, P157
Stellbrink, H-J	P238
Stellbrink, H-J	P261*
Stentarelli, C	P177
Stepchenkova, T	P085
Stephan, C	P045, P116, P188, P238
Stephens, K	O132
Sterrantino, G	P242*, P250*, P270, P280
Sterrantino, K	P254
Steuer, A	P135, P236
Stock, D	O432A, O432B
Stoeckle, M	P003, P089
Stoehr, A	P141, P157
Stöhr, W	O217
Storgaard, M	P114
Sturegård, E	P080
Su, Y-C	O422, P108, P226
Suardi, E	P183, P185*
Suay Hong, G	P092
Sued, O	P022
Suleiman, A	P078
Sulkowski, MS	O222
Sun, H-Y	O422, P108
Sun, N-L	P024
Supiot, C	P044
Surendranath, V	P174
Sutherland, R	P026*
Sutre, AF	P294
Sutton, L	P117
Sweet, D	P005*
Szetela, B	P221
Szwarcberg, J	P235, P240, P292
Szymczak, A	P221

## T

**Table:** 

Taback, N	P225
Tafazzoli, A	P073
Taka, N	P083
Tambussi, G	O112
Tambuyzer, L	P251
Tan, E	P192
Tardei, G	P093
Tarrier, L	P245*
Tasdelen Fisgin, N	P063
Tasdelen Fisgin, N	P146
Tashima, K	P240*
Tau, P	P246
Taylor, G	O133
Taylor, K	P163
Taylor, N	P135, P236
Teeranaipong, P	P208*
Tekin Koruk, S	P218
Téllez, F	P099, P281
Tellez, MJ	P255
Téllez-Molina, MJ	P287
Tempestilli, M	P057
Teppler, H	O434
Termini, R	P041, P254
Terron, A	P124
Terrón, A	P281
Thomas, N	O333
Thomas, R	P233, P283
Thompson, M	O432A, O434
Thorne, C	O133, P003, P081, P161
Thornhill, J	O112*, P288
Thornton, A	P098
Thorsteinsson, K	P114*
Tibble, J	P097
Tilley, D	P225
Timmen-Wego, M	P137
Tipple, C	P145*
Tiraboschi, J	P055*, P289
Tiraboschi, JM	P021
Tittle, V	P180
Toibaro, J	O235
Tolera Balcha, T	P080
Tomaka, FL	P240
Tommasi, C	P277, P280
Tontodonati, M	P120
Tookey, P	O133, P081, P161
Tornero, C	P102
Torregrosa, F	P065
Torrella-Domingo, A	P287
Torres, C	P282
Toutous-Trellu, L	P027
Towner, W	P235
Tozzi, V	P126
Tran-Muchowski, C	P292
Trein, A	P238
Trentalange, A	P187
Treviño-Pérez, S	O432A, O432B
Trilla Garcia, A	P299
Trinh, R	O222*
Trottier, B	P233*, P283*
Trujillo Santos, J	P105
Trunfio, M	P178
Trypsteen, W	P142, P291
Tsiganova, G	P047
Tudor, AM	P167*, P168
Tweya, H	P026

## U

**Table:** 

Unger, S	P141, P157
Ungurianu, R	P168
Uranga, A	P074, P139
Urbañska, A	P155
Urbanska, A	P221
Usó, J	P102
Uso, J	P111
Uzun Kes, N	P063, P146

## V

**Table:** 

Vaira, D	P002
Valadas, E	P195*
Valadas, E	P294*
Valantin, M-A	P249
Valencia, E	P255
Valencia, J	P228
Valencia La Rosa, JA	P278
Valenti, D	P227, P244, P266
Valero, S	P191
Valluri, S	O333
Vallvé, JC	P023, P025
Van Beckhoven, D	P002
van de Vijver, D	O154
van Delft, Y	O423A
Van den Eynde, E	P282
van den Heever, W	P170*
van den Heever-Kriek, E	P149
Van den Wijngaert, S	P002
Van Laethem, K	P222
van Lunzen, J	O153, P083
Van Ranst, M	P002
Van Wijngaerden, E	P002
van Wyk, J	P081*, P084
Vandamme, A-M	P222
Vandekerckhove, L	P002, P142, P291*
Vandercam, B	P002
Vanveggel, S	P240, P251
Vassilakis, A	P210
Vatan, R	P044
Vázquez, M	P213*
Vecchia, M	P095
Vecchiet, J	P120
Velasco Muñoz, C	P299
Vella, S	O411*
Venet, F	P140
Vento, S	P290
Ventura, F	P069, P109, P113, P156, P265
Venturini, A	P049*
Vera, C	P191, P194
Vera, F	P194
Vera-Méndez, F	P099
Vera Méndez, FJ	P105
Verbon, A	O154, P257
Verbrugge, R	P002
Vergani, B	P298
Vergara, A	P281, P282
Vergas, J	P255
Verhasselt, B	P142
Verheyen, J	P258
Verhofstede, C	P002, P291
Verine, J	P115
Vermeire, J	P142
Verolet, C	P027
Vervisch, K	P142
Vervish, K	P291
Vervoort, S	P004
Vezina, S	P233, P283
Viard, J-P	O313
Viciana, P	P281, P287
Victoria, G	P129
Viglietti, R	P013
Vignale, F	P286
Vila, A	P055, P289
Villasis Keever, A	P028*
Villasis-Keever, A	P073
Vincent, T	P014
Vinken, L	P222
Viscoli, C	P049, P186
Viskovic, K	P016, P017
Vitale, F	P079
Vjecha, MJ	O114
Vlad, D	P168
Volny-Anne, A	O215*
Volpe, A	P013
Von Braun, A	P053
von Kleist, M	P214
Vullo, V	P239, P276

## W

**Table:** 

Wainberg, M	O332*, O433*, P206
Walker, I	P275
Walker, S	O217*
Walker-Bone, K	P036
Wallace, L	P082
Walter, H	P112, P209, P211, P217, P224
Wang, C	O222
Wang, R	P096
Ward, C	P059*, P107*
Wastall, P	P096
Waters, L	P181
Weber, J	O112
Weber, R	O315, P089
Webster, D	P220
Weiss, L	O113
Wensing, A	P223
Wentworth, D	O322
Whale, R	P097
White, E	P207
Whitfield, T	P163*
Wienbreyer, A	P106, P224
Wiesmann, F	P296
Wieters, I	P116*
Wifaq, B	P117
Williams, D	P034*
Willke, A	P218
Winer, N	P171
Winston, A	P050*, P054, P256
Wirden, M	P212
Wit, F	P040*
Witak-Jêdra, M	P155
Witak-Jedra, M	P221
Witek, J	P094
Wolf, E	P238*
Wolf, T	P045, P116, P188
Wong, KH	P131*
Woods, C	P097
Wu, B-R	O422
Wu, C-H	P108, P226
Wu, P-Y	O422, P048*, P108, P226, P253
Wynne, B	P052

## X

**Table:** 

Xi, H	P084
Xu, X	O434
Xylomenos, G	P210

## Y

**Table:** 

Yakovlev, A	P062, P086
Yang, C	P264
Yang, C-J	P204
Yang, S-P	O422
Yang, S-P	P048, P253
Yao, J	P219
Yasar, K	P218
Ye, Y	P196*
Yerly, S	P213
Yfantis, V	P143
Yildirmak, T	P063, P146
Young, B	P092*, P148*
Yousef, KP	P214
Yousseff, E	P097
Yurchenko, O	P085*
Yurin, O	O236
Yýldýrmak, T	P218

## Z

**Table:** 

Zaccarelli, M	P037, P057, P123, P130, P242, P250, P270*, P277, P280
Zachry, W	P084
Zagalo, A	P294
Zakharova, N	P094
Zamora, FX	P255
Zamora, J	P268
Zanaboni, D	P013
Zanella, F	P045
Zangerle, R	P135, P236
Zanini, M	P067, P068
Zapiola, I	P172
Zavitsanou, A	P210
Zazzi, M	P258, P259
Zekan, S	P016
Zhang, J-Y	O422, P048, P253
Zhong, Y	P005
Zidovec-Lepej, S	P016
Zilmer, K	P153
Zimmermann, R	P138
Zimmermann, S	P090
Zona, S	P095, P121, P128, P177, P184*
Zong, J	P052
Zoufaly, A	P083*
Zucman, D	P070*
Zurawski, C	P273

